# Constructing Molybdenum Phosphide@Cobalt Phosphide Heterostructure Nanoarrays on Nickel Foam as a Bifunctional Electrocatalyst for Enhanced Overall Water Splitting

**DOI:** 10.3390/molecules28093647

**Published:** 2023-04-22

**Authors:** Yingchun Huang, Hongming Chen, Busheng Zhang

**Affiliations:** Shunde Innovation School, University of Science and Technology Beijing, Foshan 528399, China

**Keywords:** bifunctional electrocatalyst, overall water splitting, heterostructure, self-supporting

## Abstract

The construction of multi-level heterostructure materials is an effective way to further the catalytic activity of catalysts. Here, we assembled self-supporting MoS_2_@Co precursor nanoarrays on the support of nickel foam by coupling the hydrothermal method and electrostatic adsorption method, followed by a low-temperature phosphating strategy to obtain Mo_4_P_3_@CoP/NF electrode materials. The construction of the Mo_4_P_3_@CoP heterojunction can lead to electron transfer from the Mo_4_P_3_ phase to the CoP phase at the phase interface region, thereby optimizing the charge structure of the active sites. Not only that, the introduction of Mo_4_P_3_ will make water molecules preferentially adsorb on its surface, which will help to reduce the water molecule decomposition energy barrier of the Mo_4_P_3_@CoP heterojunction. Subsequently, H* overflowed to the surface of CoP to generate H_2_ molecules, which finally showed a lower water molecule decomposition energy barrier and better intermediate adsorption energy. Based on this, the material shows excellent HER/OER dual-functional catalytic performance under alkaline conditions. It only needs 72 mV and 238 mV to reach 10 mA/cm^2^ for HER and OER, respectively. Meanwhile, in a two-electrode system, only 1.54 V is needed to reach 10 mA/cm^2^, which is even better than the commercial RuO_2_/NF||Pt/C/NF electrode pair. In addition, the unique self-supporting structure design ensures unimpeded electron transmission between the loaded nanoarray and the conductive substrate. The loose porous surface design is not only conducive to the full exposure of more catalytic sites on the surface but also facilitates the smooth escape of gas after production so as to improve the utilization rate of active sites. This work has important guiding significance for the design and development of high-performance bifunctional electrolytic water catalysts.

## 1. Introduction

Energy shortage and environmental pollution are problems facing mankind today. The development of clean energy and the exploration of green synthesis methods are the means to solve these problems. Due to its high calorific value of combustion and cleaning product (H_2_O), hydrogen is the ideal clean energy in the future. Water electrolysis is an effective method for hydrogen production [1,2]. Hydrogen production from electrolysis water is the decomposition reaction of water under a certain voltage, which consists of OER and HER two half reactions. Due to the slow reaction kinetics, there is usually an overpotential (the required potential is higher than the water decomposition potential of 1.23 V) [3,4]. This limits the development of electrocatalytic water decomposition to a great extent.

Catalysts can effectively reduce overpotential, but most of the catalysts with excellent performance are noble metals, such as RuO_2_, Pt, etc., and the characteristics of high price and scarce reserves hinder their large-scale application. Therefore, the research and development of efficient and cheap non-noble metals-based catalysts have become a current research hotspot [5,6,7,8]. Among them, transition metal compounds, such as phosphides [9], oxides [10,11], and sulfides [12], have attracted wide attention due to their low cost, high oxidation and reduction activity, and abundant reserves. Cobalt-based phosphides showed highly efficient and stable HER properties [13,14]. However, phosphides show the poor catalytic performance of OER [15]. Despite recent progress, few cobalt-based phosphides can efficiently perform HER, OER, and overall water splitting at the same electrolyte.

The construction of heterojunction has been proven to be a common and effective method to improve electrocatalytic activity [16,17]. By forming a heterogeneous structure, the electron configuration of the heterogeneous interface can be effectively adjusted in order to enhance the charge transfer ability, overcome the high catalytic reaction barrier, reduce the HER and OER energy barrier, and improve the intrinsic activity [18,19]. Sun et al. synthesized the flower-like Co@CoP_2_ heterostructures grown on copper foam under optimal conditions by adjusting the concentration of phytic acid. The transfer of electrons from CoP_2_ to the Co side of the metal can be observed through theoretical model calculations, causing charge accumulation, and the relatively enriched electron state can promote the rapid reaction kinetics of HER and OER [20]. Therefore, the heterogeneous structure can effectively regulate the interfacial charge, enhance the electron transfer and reduce the charge transfer impedance.

The maximum utilization of a heterogeneous interface can provide full play to the overall performance of the catalyst. Hence, by virtue of the characteristics of small particle size and large specific surface area, small-sized nanoparticles have more active sites, so they can adsorb reactant molecules more effectively and promote the catalytic reaction [21,22]. Lee et al. developed a binderless spinel oxide nanoparticle-coated nickel foam as an efficient electrocatalyst for water oxidation. The optimized CoFe_2_O_4_ nanoparticles have more active catalytic sites, better charge transfer, stronger ion diffusion, and suitable reaction kinetics [23].

We prepared Ni foam-supported Mo_4_P_3_@CoP heterojunction nanowire arrays (Mo_4_P_3_@CoP/NF) by combining hydrothermal, electrostatic adsorption, and phosphating methods. Compared with nanoparticles, nickel foam loading can save the step of preparing electrode ink and shorten the catalyst preparation cycle; the three-dimensional array structure can expose more sites in contact with the electrolyte and increase the reaction site; Mo_4_P_3_@CoP heterojunction can optimize the electronic structure, accelerate the transfer of electrons from Mo_4_P_3_ to CoP, and promote the improvement of catalytic performance. As a bifunctional catalyst, Mo_4_P_3_@CoP/NF exhibits an overpotential of 283 mV for OER and 72 mV for HER at a current density of 10 mA/cm^2^ under alkaline conditions. The material has excellent application prospects as a catalyst for the electrolysis of water.

## 2. Results and Discussion

### 2.1. Characterization

**Figure 1:**
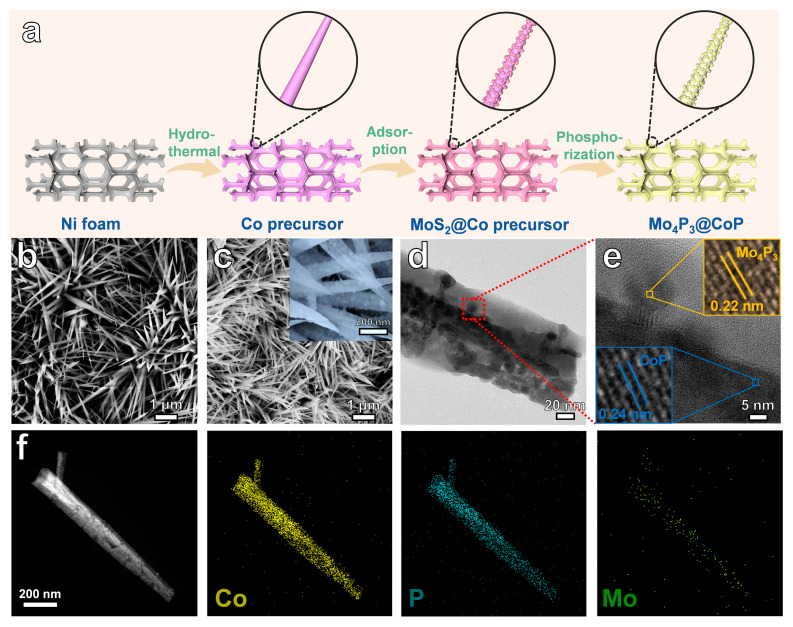
(**a**) Schematic illustration of the preparation of Mo_4_P_3_@CoP nanoarrays on Ni foam (NF). Scanning electron microscopy (SEM) images of the (**b**) MoS_2_@Co precursor/NF, (**c**) Mo_4_P_3_@CoP/NF. (**d**) Transmission electron microscopy (TEM) image of Mo_4_P_3_@CoP nanowire. (**e**) HRTEM image of Mo_4_P_3_@CoP nanowire from the region (**d**) in the panel. (**f**) High-angle annular dark field scanning TEM (HAADF-STEM) image of single Mo_4_P_3_@CoP and the corresponding EDS mapping for elements of Co, P, and Mo.

Figure 1a shows the synthesis route of bimetallic phosphide heterostructures with nickel foam as self-supporting CoP nanowires loaded with Mo_4_P_3_ nanoparticles (NPs). The detailed preparation process can be found in the experimental part. The crystal structure, morphology, and elemental distribution of the sample are demonstrated by X-ray diffraction (XRD), field emission scanning electron microscopy (SEM), and transmission electron microscopy (TEM). Firstly, Co precursor nanowires were grown on foamed nickel by the hydrothermal method, and its chemical composition could be fitted in the XRD pattern of Co(CO_3_)_0.5_(OH)_0.11_·H_2_O (Figure S1). Figure S2 shows a uniformly covered nanowire morphology with particle sizes in the range of 50~200 nm. The SEM image shows it is in sharp contrast with the smooth nickel foam in Figure S3. At the same time, the protruding and relatively large 1D nanowire structure can expose a larger specific surface area, which can not only fully contact with the electrolyte and improve the reaction efficiency but also provide more attachment sites for the subsequent MoS_2_ NPs, thereby increasing the number of interfaces and enhancing the efficiency of the reaction. As shown in Figure 1b, the MoS_2_ NPs were uniformly and densely distributed on the surface of the Co precursor nanowires after soaking for a long time. Finally, the heterogeneous bimetallic phosphide heterostructure Mo_4_P_3_@CoP/NF was obtained by further phosphating treatment. In Figure 1c, the morphology of the sample before and after phosphating is compared, the nickel foam and the nanowire structure loaded with NPs are not damaged, and the overall morphology remains unchanged. However, through high-rate SEM, it can be observed that there are some rough and granular feelings on the surface, which may be caused by phosphating (Figure 1c inset). It shows that the self-supporting structural design has suitable stability and can maintain satisfactory contact between the substrate and the support, thereby improving the electron transport and mass transfer capabilities during the catalytic reaction, as well as enhancing the tolerance and long-term stability of the catalyst. The TEM image of Figure 1d shows that the Mo_4_P_3_ NPs are uniformly grown on the surface of the CoP nanowires, their sizes are mainly distributed between 5 and 10 nm, and the small-sized NPs have more abundant surface-active sites, thus promoting the reaction process. Figure 1e shows the interplanar spacing of 0.24 nm and 0.22 nm of nanowire CoP and NPs-loaded Mo_4_P_3_, which belongs to the (111) plane of CoP and the (116) plane of Mo_4_P_3_, respectively. It is consistent with the crystal plane analysis of selected electron diffraction (Figure S4). In addition, the STEM analysis of Figure 1f shows that the Co element is mainly distributed in the middle part of the nanowires, while Mo is distributed near the surface edge of the nanowires, which is consistent with the actual structural composition analysis.

**Figure 2:**
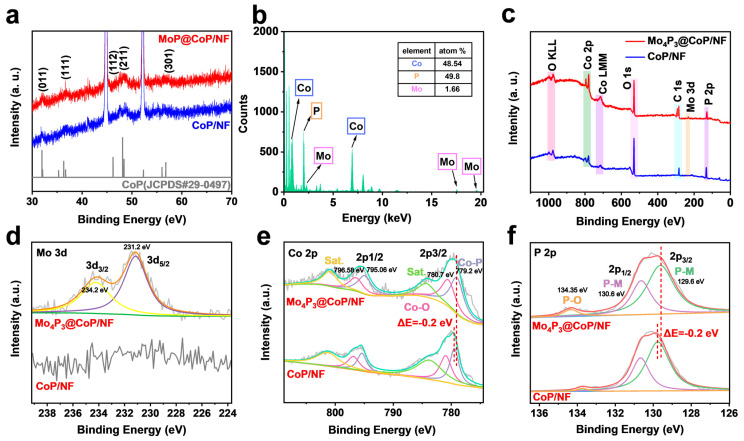
(**a**) XRD patterns, (**b**) full XPS spectra of Mo_4_P_3_@CoP/NF and CoP/NF. High−resolution (**c**) Mo 2p, (**d**) Co 2p, and (**e**) P 2p XPS spectra of Mo_4_P_3_@CoP/NF and CoP/NF. (**f**) Mo_4_P_3_ and CoP work function step diagrams.

In order to further investigate the electron modulation effect at the interface between Mo_4_P_3_ NPs and CoP nanowires, X-ray diffraction (XRD) and X-ray photoelectron spectroscopy (XPS) were used to characterize Mo_4_P_3_@CoP-NF samples and to study the binding energies of the material components and valence electrons, respectively. The XRD pattern of the Mo_4_P_3_@CoP/NF sample is shown in Figure 2a, where the peaks at 31.6° and 56.8° correspond to the (011) and (211) planes of CoP (JCPDS No. 29-0497), respectively. The peaks at 44.8°, 52.2°, and 76.7° correspond to the nickel elemental of the base nickel foam. Similar to the XRD test results of MoS_2_@Co precursor/NF during the synthesis process (Figure S5), only a very small amount of Mo_4_P_3_ NPs (1.66%) is needed to build a uniform and appropriate interface structure on the surface of CoP nanowires (Figure 2b). In addition, the size of NPs (about 10 nm) is extremely small, and it is difficult to clearly observe the diffraction peaks of Mo_4_P_3_ NPs in the XRD mode of Mo_4_P_3_@CoP/NF. The XRD test results are consistent with expectations, but they are not enough to intuitively reflect the interaction between CoP nanowires and Mo_4_P_3_ NPs [24,25]. Therefore, we used the XPS test to find the interaction between them. The test results show that there are obvious Co 2p peaks and P 2p peaks in the full XPS spectrum (Figure 2c). Compared with single-phase CoP nanowires, Mo_4_P_3_@CoP has slight fluctuations in the range of Mo 3d. In order to analyze the transfer direction of electrons at the interface, after performing multiple linear regression fitting on the high-resolution fine spectrum, the binding energy changes of each element were further compared and analyzed with or without Mo_4_P_3_ NPs loading. Although the amount of Mo_4_P_3_ NPs in Mo_4_P_3_@CoP is small, compared with single-phase CoP nanowires, the signal of Mo in Mo_4_P_3_@CoP is still obvious in the high-resolution fine spectrum of Mo 3d because the Mo_4_P_3_ NPs are loaded in on the surface of nanowires, and X-rays can effectively excite valence electrons in Mo_4_P_3_ NPs. A set of spin-orbit splitting peaks at 234.20 and 231.20 eV shown in Figure 2d belong to Mo 3d_3/2_ and Mo 3d_5/2_ peaks on the NPs. Figure 2e shows that in the Co 2p spectrum, the signals of a set of spin-orbit splitting peaks at 779.20/795.06 eV originate from Co 2p_3/2_ and Co 2p_1/2_ in Co-P [26,27]. A group of peaks at 780.7 and 796.58 eV are the signals of Co 2p_3/2_ and Co 2p_1/2_ of the Co-O bond caused by surface oxidation of the material, and a group of satellite peaks at 784.45 and 800.95 eV [28,29]. Compared with the Co 2p high-resolution fine spectrum of single-phase CoP nanowires without Mo_4_P_3_ NPs, the binding energy of the corresponding peaks of Mo_4_P_3_@CoP has a shift of −0.2 eV. Coincidentally, a similar shift also appears in the high-resolution fine spectrum of P 2p. As shown in Figure 2f, the signals of a set of spin-orbit splitting peaks at 129.60 and 130.60 eV originate from P 2p_3/2_ and P 2p_1/2_ in metal phosphides, and the broad peak signal at 134.35 eV belongs to phosphate caused by air oxidation species [30]. Compared with single-phase CoP nanowires, the corresponding peaks in Mo_4_P_3_@CoP move −0.2 eV, respectively. Electron transfer phenomena in Co 2p and P 2p may be due to the modulation of electronic structure in the interfacial region caused by the interaction between CoP and Mo_4_P_3_ lattice, which may be conducive to the enhancement of catalytic performance.

### 2.2. HER Performance

**Figure 3:**
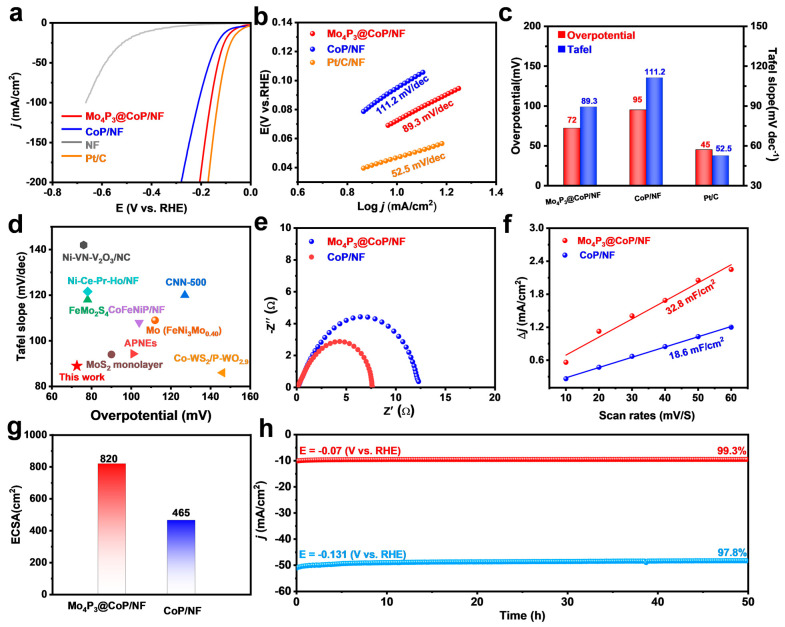
(**a**) Polarization curves of Mo_4_P_3_@CoP/NF, CoP/NF, NF, and Pt/C. (**b**) Tafel curves of Mo_4_P_3_@CoP/NF, CoP/NF and Pt/C. (**c**) Comparison chart of overpotential and Tafel slope. (**d**) HER performance comparison of the Mo_4_P_3_@CoP/NF with different catalysts published recently. (**e**) Electrochemical impedance of Mo_4_P_3_@CoP/NF, CoP/NF. (**f**) ECSA values of C_dl_. (**g**) Mo_4_P_3_@CoP/NF and CoP/NF. (**h**) I−t curves at −0.07 V vs. RHE and −0.0131 V vs. RHE, respectively.

In order to verify that the modulation of the electronic structure in the interfacial region caused by the interaction of CoP and Mo_4_P_3_ lattice is beneficial to the enhancement of catalytic performance, we conducted electrochemical tests on Mo_4_P_3_@CoP/NF and control samples with graphite rod as the counter electrode and Hg/HgO electrode as the reference electrode in the alkaline environment of 1 M KOH solution. We tested the catalytic activity enhancement of these samples in the potential range of −0.9~−1.8 V vs. Hg/HgO. As shown in Figure 3a, to achieve a current density of 10 mA/cm^2^, the Mo_4_P_3_@CoP/NF electrode requires only 72 mV, significantly lower than CoP/NF (95 mV), NF (410 mV), and only higher than the commercial Pt/C/NF electrode (45 mV). Next, we calculated the Tafel slope. The smaller the value of the Tafel slope, the smaller the overpotential that can be used to achieve a certain current density. This means that the charge transfer kinetics are faster. The Tafel slope can be measured by three different methods, mainly including (1) direct measurement by electrochemical workstation, (2) conversion by linear sweep voltammetry (LSV) curve, and (3) fitting by EIS data. In this work, the Tafel slope value is obtained by formula conversion of LSV curve data. The Tafel slope of the Mo_4_P_3_@CoP/NF electrode was found to be 89.3 mV/dec, only slightly higher than the Pt/C/CF electrode (52.5 mV/dec) (Figure 3b). However, it is much smaller than single-phase CoP/CF (111.2 mV/dec). We compared the activities of different catalysts by comparing the Tafel slope and overpotential at constant current density (Figure 3c). The electrodes were compared with catalysts reported in other studies, showing higher activity than most electrodes reported in previous studies; specific data can be found in Figure 3d. In addition, electrochemical impedance spectroscopy (EIS) is an evaluation index for analyzing the kinetics of electrocatalysts and electrolytes and the internal electron transfer laws of electrode materials. Typically, the diameter of the semicircle represents the charge transfer resistance (R_ct_). The smaller the value of R_ct_, it indicates that the catalyst has faster electron transport speed, better electrical conductivity, and thus has more prominent electrocatalytic performance. So we performed the EIS test. The frequency range of the EIS test is 0.1~100 kHz, and the voltage is −0.1 V vs. RHE. As shown in Figure 3e, the Mo_4_P_3_@CoP/NF electrode exhibits the most efficient electron transfer ability in HER electrochemical catalysis, and its charge transfer resistance (R_ct_) is significantly smaller than that of other electrodes [31,32]. CV tests shown in Figure S6 were performed using a scan rate of 10~60 mV/s. Through CV, we calculated the C_dl_ value of the electrode in Figure 3f. The C_dl_ value of the Mo_4_P_3_@CoP/NF electrode was 32.8 mF/cm^2^, which was significantly higher than CoP/NF (18.6 mF/cm^2^). At the same time, the Mo_4_P_3_@CoP/NF electrode also possessed the largest electrochemically active surface area (ECSA) value among these prepared electrodes, with an ECSA of 820 cm^2^ for Mo_4_P_3_@CoP/NF and 465 cm^2^ for CoP/NF (Figure 3g). To further determine the influence of the construction of composite structures on the specific surface area of materials, we performed the Brunauer–Emmett–Teller (BET) characterization of Mo_4_P_3_/CoP/NF and single-phased CoP/NF. As exhibited in Figure S7, the calculated specific surface area of Mo_4_P_3_/CoP/NF is 269.86 m^2^/g, which is much bigger than single-phased CoP/NF (224.89 m^2^/g). The larger surface area is conducive to exposing abundant superficial active sites in the reaction process and ensuring significantly promoted electrocatalytic performance. These results indicate that the preparation of the heterointerface has a great influence on enhancing the intrinsic electron transport rate, central site reactivity, and the number of surface-active sites in the electrode [33]. In addition, stability tests were performed at −0.07 and −0131 V vs. RHE potentials, and the results showed (Figure 3h) that the activity retention of the HER catalyst was still high in 1 M KOH solution, reaching 99.3%, and 97.8%, respectively. Moreover, the LSV curve after the I-t test has no obvious activity decrease compared with that before the test, which indicates that it has excellent catalytic durability in an alkaline system (Figure S8). To assess the stability of the material, various characterization techniques, including XRD, SEM, and TEM, were employed to analyze possible changes in the complex electrolysis process in chemical composition and structure. In Figure S9, the XRD results show that after HER treatment, the main components of the electrode are relatively stable without significant changes, showing suitable compositional stability. Furthermore, the SEM and TEM images shown in Figure S10 illustrated that the nanostructures are still intact, indicating excellent morphological stability. In addition, we carried out the XPS test on the sample after HER, and the results showed that the valence states of the elements in the sample before and after HER did not change significantly (Figure S11). In addition, we also checked the metal leaking amount of composite Mo_4_P_3_/CoP/NF and the single-phased CoP/NF during stability tests. We further performed ICP−OES tests of the two samples, and the detailed results are shown in Tables S1 and S2. Moreover, we plotted the dissociation content at different times, as displayed in Figure S12. Specifically, the Co dissociation rate of Mo_4_P_3_/CoP/NF is 8.1 ng/h, which is significantly decreased compared to single-phased CoP/NF (14.8 ng/h). Meanwhile, the Mo dissociation rate of Mo_4_P_3_/CoP/NF is as low as 5.2 ng/h. These results indicate that the strong interaction of Mo_4_P_3_ and CoP is beneficial to stabilize the superficial active metal sites for lower dissociation rate and enhanced catalytic stability.

### 2.3. Mechanism Study

**Figure 4:**
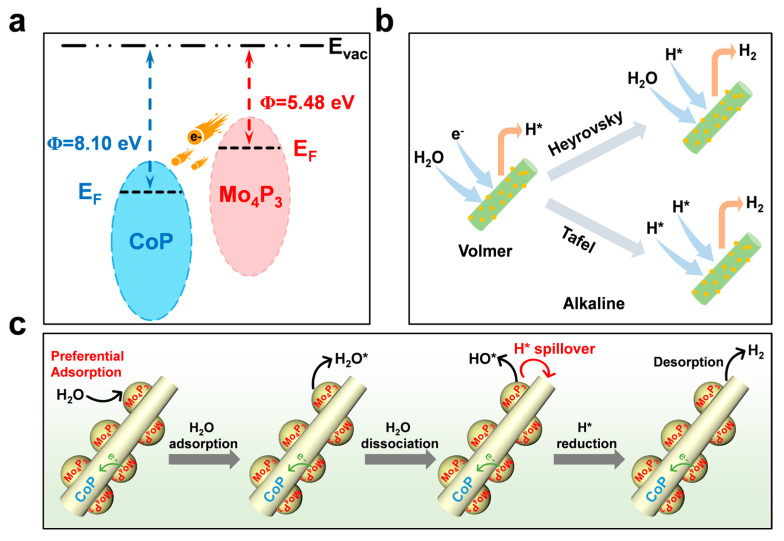
(**a**) Mo_4_P_3_ and CoP work function step diagrams. (**b**) Schematic diagram of HER steps for alkaline conditions. (**c**) HER reaction process of Mo_4_P_3_@CoP/NF under alkaline conditions.

In order to explore the HER catalytic mechanism of Mo_4_P_3_@CoP materials, according to previous work, we obtained that the work function of CoP materials is 8.1 eV, which is significantly higher than 5.48 eV of Mo_4_P_3_ (Figure 4a) [34,35]. This enables electron transfer between the CoP nanowires and the Mo_4_P_3_ NPs, and the electrons are transferred from Mo_4_P_3_ to CoP until equilibrium is reached. Finally, due to the low electrostatic potential of Mo_4_P_3_, electron loss occurs in Mo_4_P_3_ while electron gain occurs in CoP. The hydrogen evolution reaction (HER) process undergoes two different reaction mechanisms in alkaline media, as shown in Figure 4b [36]:

H_2_O + e^−^ + * → H* + OH^−^, Volmer step (1)

H* + H* → H_2_, Tafel step (2)

H* + H_2_O + e^−^ → H_2_ + OH^−^, Heyrovsky step (3)

As mentioned above, in the alkaline electrolyte environment, it is divided into two steps: the Volmer step and the Tafel step or the Heyrovsky step [37,38]. In alkaline environments, due to the existence of a large number of hydroxide ions, it is difficult to directly adsorb H ions, so water is often required to dissociate first in order to produce abundant H ions. Therefore, small-size Mo_4_P_3_ nanoparticles can contact electrolytes to a large extent so as to fully carry out hydrolytic dissociation reactions to produce a large number of H ions, which contributes to subsequent adsorption and desorption processes. In addition, the vertically grown nanowires can not only further increase the contact area of Mo_4_P_3_ nanoparticles with the electrolyte but also contribute to the hydrogen gas precipitation and then solve the problem of gas blockage hindering the catalytic reaction, thus promoting the efficient kinetic reaction [39].

In view of the above studies, we found that the Mo_4_P_3_@CoP heterostructure nanoarray material exhibited significantly enhanced HER electrocatalytic activity. For the HER process in alkaline media, the water molecule decomposition process and the H* adsorption process together restrict the overall energy consumption of the reaction. For CoP single-phase catalysts, it has been proven to be a material with excellent H* adsorption energy [40]. After introducing the Mo_4_P_3_ phase to construct the Mo_4_P_3_@CoP heterointerface, the HER activity is significantly improved. Therefore, we believe that after the introduction of the Mo_4_P_3_ phase, the water molecules are more likely to dissociate into H* and OH*; that is, the water molecule decomposition energy barrier will be significantly reduced. So, we believe that for the Mo_4_P_3_@CoP heterojunction, during the alkaline HER process, water molecules will preferentially adsorb on the Mo_4_P_3_ surface and easily decompose into H* and OH* [41], and then the generated H* will be transferred to the CoP surface sites through the overflow process, it is, because of its better H* adsorption energy, and then the H* combines with each other to generate H_2_ molecules that dissociate from the CoP surface (Figure 4c) [42]. In summary, the synergistic effect of Mo_4_P_3_ and CoP ensures that the catalyst exhibits significantly improved HER catalytic performance.

### 2.4. OER Performance

**Figure 5:**
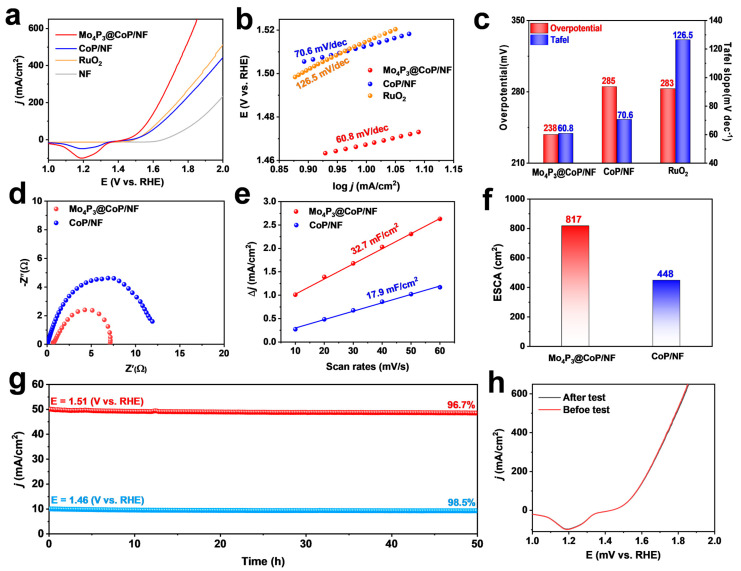
(**a**) Polarization curves of Mo_4_P_3_@CoP/NF, CoP/NF, NF, and RuO_2_. (**b**) Tafel curves of Mo_4_P_3_@CoP/NF, CoP/NF, and RuO_2_. (**c**) Comparison chart of overpotential and Tafel. (**d**) Electrochemical impedance spectra of Mo_4_P_3_@CoP/NF, CoP/NF. (**e**) Double-layer capacitor (C_dl_). (**f**) ECSA values of Mo_4_P_3_@CoP/NF and CoP/NF. (**g**) I−t plots at 1.51 V vs. RHE and 1.46 V vs. RHE. (**h**) LSV plots before and after the I-t test.

In addition to testing the catalytic activity of HER, we also evaluated the performance of OER. Similarly, the polarization curve current density (J) versus applied potential (V vs. RHE) was obtained by LSV. In Figure 5a, the LSV curves of the catalyst Mo_4_P_3_@CoP/NF in 1.0 M KOH solution, where only an overpotential (ƞ) of 238 mV is needed to achieve a current density of 10 mA/cm^2^. At the same time, the performance tests of the comparative samples CoP/NF and RuO_2_/NF were also performed. At the current density of 10 mA/cm^2^, the overpotentials were 283 mV and 285 mV, respectively. On the other hand, the catalytic contribution of bare nickel foam (NF) to OER is very low, which indicates that the existence of Mo_4_P_3_@CoP electrocatalyst on the electrode surface can effectively enhance the catalytic activity of OER. The specific OER process is relatively complicated and has not yet been definitively determined. The widely accepted reaction mechanism is the adsorbate evolution mechanism (AEM) [43,44]. The concrete reaction process is as follows:

OH + * → HO* + e^−^ (4)

HO* → O* + e^−^ + H^+^ (5)

O* + OH → HOO* + e^−^ (6)

HOO* → * + O_2_ + H^+^ + e^−^ (7)

The presence of electrocatalysts on the anode electrodes can greatly reduce the overpotential of the OER and facilitate the charge transfer between electrodes and electrolytes [45]. The Tafel slope is an important parameter to evaluate the intrinsic activity of a catalyst in a specific catalytic reaction. Further selecting the linear part from the LSV curve, we draw the Tafel plot between the potential (V) and the logarithm of the current density (mA/cm^2^), where the minimum Tafel slope value indicates more efficient charge transfer and higher catalytic activity. As shown in Figure 5b, the Tafel slope of Mo_4_P_3_@CoP/NF (60.8 mV/dec) is lower than that of CoP/NF (70.6 mV/dec) and RuO_2_/NF (98.7 mV/dec) electrodes. It is further shown that the Mo_4_P_3_@CoP/NF catalyst can enhance the adsorption of intermediates and effectively reduce the overpotential of the corresponding catalytic half-reaction, accelerating the OER process. Thus, the activities of different catalysts can be compared by comparing the Tafel slope and overpotential at constant current density (Figure 5c). Electrochemical impedance spectroscopy (EIS) plays a vital role in the study of catalyst performance and can represent the charge transfer ability of different catalysts. According to the equivalent circuit established according to the Nyquist mode (Figure 5d), Mo_4_P_3_@CoP/NF has suitable conductivity, and CoP/NF electron transfer resistance (R_ct_) increases, indicating a higher charge transfer barrier and lower performance. The CV curves of Mo_4_P_3_@CoP/NF and CoP/NF at different scan rates (10~60 mV/s) in the non-Faradaic region are shown in Figure S13. The underlying formula Δj/2 = (j_anodic_ − j_cathodic_)/2 yields a curve versus scan rate in Figure 5e. The C_dl_ values of Mo4P3@CoP/NF and CoP/NF are 32.7 mF/cm^2^ and 17.9 mF/cm^2^, respectively. The ECSA value was further calculated (Figure 5f), and it was concluded that Mo_4_P_3_@CoP/NF had the largest active surface area value (817 cm^2^) compared to CoP/NF (448 cm^2^). Mo_4_P_3_@CoP/NF has a larger active surface area and more exposed active sites, and thus, the samples obtain higher OER catalytic activity. As shown in Figure 5g, the excellent stability of the sample Mo_4_P_3_@CoP/NF was further verified by chronopotentiometry, and the activity retention rate could reach 98.5% and 96.7% after continuous operation at different voltages of 1.46 V and 1.51 V for 50 h, respectively. Then in a three-electrode system, we investigated the OER catalytic performance of Mo_4_P_3_@CoP/NF after electrical stability tests by LSV. As shown in Figure 5h, the LSV curve after the stability test has no significant deviation compared with that before the test, indicating that Mo_4_P_3_@CoP/NF has high catalytic performance before and after the stability test and has a certain potential for industrial application. To further understand the composition and morphology as well as valence changes of the samples after the long-term stability test, XRD, SEM, and XPS characterization were carried out. In Figure S14, it can be observed that some changes occurred on the sample surface before and after the OER test. As shown in Figure S15, it can be seen in the XRD patterns that the diffraction peaks of each phase of CoP are preserved. However, through the XPS test of samples after OER, it was found that the proportion of the Co−O bond increased, and the binding energy of the Co element increased. We guessed that the CoP on the surface of the sample transformed into an amorphous CoOOH during the OER process (Figure S16) [46]. In conclusion, the catalyst Mo_4_P_3_@CoP/NF has outstanding long-term stability and catalytic activity.

### 2.5. Overall Water-Splitting Performance

**Figure 6:**
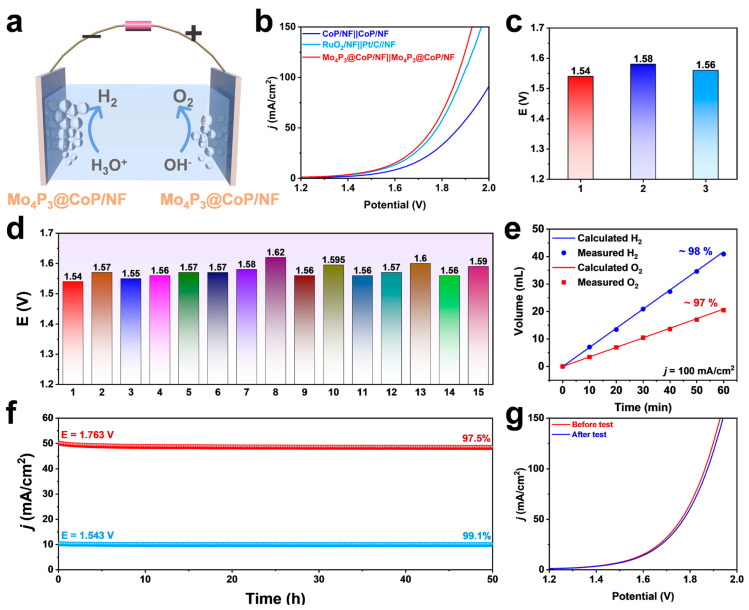
(**a**) Schematic diagram of the electrolyzer for overall water splitting. (**b**) In 1.0 M KOH solution, Mo_4_P_3_@CoP/NF||Mo_4_P_3_@CoP/NF, CoP/NF||CoP/NF, and RuO_2_/NF||RuO_2_/NF are fully hydrolyzed polarization curves and (**c**) voltage at 10 mA/cm^2^. (**d**) Performance comparison of Mo_4_P_3_ @CoP/NF with recently reported catalysts. (**e**) Faradaic efficiency of electrolytic water remeasured by drainage method. (**f**) At constant voltages of 1.543 V and 1.763 V, the I−t curve of Mo_4_P_3_@CoP/NF. (**g**) LSV images of Mo_4_P_3_@CoP/NF before and after the stability test.

Mo_4_P_3_@CoP/NF exhibits excellent catalytic performance when used as a catalyst for HER and OER. To research the catalytic performance of Mo_4_P_3_ NPs heterostructure for overall water splitting, a double-layer catalyst using Mo_4_P_3_@CoP/NF as both anode and cathode was designed. Electrode electrolyzer and evaluated in an alkaline environment (Figure 6a). To compare their overall water-splitting capabilities, CoP/NF||CoP/NF and noble metal Pt/C/NF||RuO_2_/NF electrolyzers with the same mass loading were also assembled and tested under the same conditions. As shown in Figure 6b, Mo_4_P_3_@CoP/NF||Mo_4_P_3_@CoP/NF exhibits remarkably suitable catalytic performance. The control samples of CoP/NF||CoP/NF and Pt/C/NF||RuO_2_/NF require voltages of 1.58 V and 1.56 V to achieve a current density of 10 mA/cm^2^, while Mo_4_P_3_@CoP/NF||Mo_4_P_3_@ CoP/NF only needs 1.54 V to reach the same effect (Figure 6c). What is more, compared with other recently reported catalysts, it can be found that it has excellent catalytic performance for overall water splitting, indicating that it has great potential to develop into a high-performance catalyst (Figure 6d). The overall water-splitting voltage of the catalyst is certainly one of the indicators for evaluating the performance, but due to some environmental restrictions in practical applications, we must consider the hydrogen generation efficiency of the catalyst. As shown in Figure 6e, the faradaic efficiencies of hydrogen and oxygen of Mo_4_P_3_@CoP/NF are about 98% and 97%, respectively, in the overall water splitting, indicating that the catalyst can save much energy loss in industrial applications and ensure energy conservation. In addition to having satisfactory catalytic performance and high catalytic efficiency, the stability of material samples is also a factor that must be considered. The I-t curve test of the sample is shown in Figure 6f. It still has a stable current density after 50 h at high voltages of 1.543 V and 1.763 V, and the LSV curve before and after the test basically does not change (Figure 6g). This is a closer illustration that it has excellent catalytic durability and mechanical stability, which is mainly due to the structural stability of the self-supporting design, which ensures a tight connection between the active materials and the electrode.

The reason why Mo_4_P_3_@CoP/NF||Mo_4_P_3_@CoP/NF has such excellent electrochemical activity in the application of overall water splitting is mainly a result of the following two aspects: firstly, the heterostructure design between Mo_4_P_3_ and CoP has a high degree of crystallinity. Therefore, the interface has a strong electronic interaction, which can effectively adjust the electron distribution between the two phases. It can not only accelerate the electron transfer in the catalytic reaction but also optimize the water dissociation energy [47,48,49]. Moreover, due to the interfacial effect, more abundant active sites will be generated to enhance the catalytic activity. Secondly, the in situ growth on NF strengthens the tight connection between the active materials and the electrodes, improves the structural stability and tolerance of the nanoarray, and enables the active material to play its role to the greatest extent. Moreover, NF can provide a larger surface area to grow active species, thus exposing more active sites. The self-supporting structure design can avoid the use of binders, thus improving the conductivity of the electrode, accelerating the charge transfer between active materials, and promoting the rapid progress of the reaction [50,51,52]. Therefore, Mo_4_P_3_@CoP/NF, with the self-supporting structure design and interface engineering optimization, has broad application prospects in energy conversion and optimization.

## 3. Materials and Methods

### 3.1. Synthesis of Co(CO_3_)_0.5_(OH)_0.11_·H_2_O Nanowires on Nickel Foam

Co(CO_3_)_0.5_(OH)_0.11_·H_2_O nanowires were prepared by the hydrothermal method in the wet chemical method. Firstly, the cut foam was pretreated with diluted hydrochloric acid, deionized water, and ethanol in sequence and then ultrasonicated for 20 min, respectively. Next, we prepared 35 mL of the deionized aqueous solution containing 0.582 g Co(NO_3_)_2_·6H_2_O, 0.222 g NH_4_F, and 0.721 g CO(NH_2_)_2_ and transferred it to a 50 mL Telfon−lined autoclave. Then, we immersed the pretreated nickel foam in it and put it in an oven at 120 °C for 6 h. After the reaction was completed and cooled to room temperature, it was taken out and rinsed with deionized water and ethanol, respectively. Finally, it was vacuum dried and stored for subsequent reactions.

### 3.2. Preparation of MoS_2_ NPs Dispersion

The MoS_2_ NPs solution was also prepared by a simple hydrothermal method. We took 0.125 g NaMoO_4_·2H_2_O and put it into 12.5 mL ultrapure water and performed magnetic stirring and ultrasonic treatment to dissolve it completely. Then, we slowly added 0.1 mol/L dilute hydrochloric acid solution to adjust the pH value to 6.5. We weighed 0.215 g of glutathione (GSH) and dissolved it into 25 mL of ultrapure water, mixed it with the above homogeneous solution, sonicated, and stirred it for 10 min, then transferred it to a 50 mL Telfon-lined autoclave. We put the reaction kettle into a preheated oven at 200 °C. After reacting for 24 h, we removed the large particles generated during the reaction by centrifugation and stored the obtained yellow supernatant in a dark environment at 4 °C.

### 3.3. Preparation of MoS_2_@Co Precursor/NF

MoS_2_@Co precursor/NF was prepared by immersion adsorption at room temperature. The nickel foam with Co precursor growth was placed in a certain amount of MoS_2_ NPs solution and soaked for 10 min, and then it was taken out and rinsed with deionized water and ethanol. It was dried overnight in a vacuum oven.

### 3.4. Preparation of Mo_4_P_3_@CoP/NF

Synthesis of metal phosphides was prepared by one-step phosphating in a tube furnace. We weighed 0.5 g NaH_2_PO_2_·H_2_O and placed the previously synthesized MoS_2_@Co precursor/NF in two porcelain boats, respectively, with the two placed side by side and the former located upstream. After flowing an Ar gas for 30 min, it was heated to 300 °C at a heating rate of 2 °C/min and kept in the Ar gas environment for 2 h. Upon cooling to room temperature, the obtained Mo_4_P_3_@CoP/NF sample was taken out. CoP/NF can also be obtained through a similar reaction process; just replace MoS_2_@Co precursor/NF with Co precursor/NF.

### 3.5. Preparation of Pt/C and RuO_2_ Electrodes

A total of 20 mg of Pt/C (20%) was uniformly dispersed in a solution of deionized water, ethanol, and isopropanol at a ratio of 1:1:1 and ultrasonicated for 40 min. Afterward, 200 μL of Pt/C dispersion was applied to the nickel foam (1 × 1 cm^2^) and dried at 60 °C. The above operation was repeated twice to obtain Pt/C with an average mass loading of ~3.8 mg/cm^2^. We replaced Pt/C with RuO_2_ and used the same method to obtain RuO_2_/NF electrode with the same mass loading.

### 3.6. Material Characterization

The morphology, particle size, and element distribution of the samples were tested and analyzed by field emission scanning electron microscope (SUPAR 55) and transmission electron microscope (JOEL JEM-2200FS), respectively. The crystal structure characteristic information of the material was obtained by X-ray diffraction (Rigaku Dmax 2200). The electronic structure states of the elements of the samples were collected by X-ray photoelectron spectroscopy (PHI 5000 Versaprobe III) using Al Kα as the X-ray source.

### 3.7. Electrochemical Test

The electrochemical performance of the samples was tested using an electrochemical workstation model CHI760E. The active material was used as the working electrode, the saturated Hg/HgO electrode was used as the reference electrode, and the graphite rod was used as the counter electrode. A typical three-electrode system was used to test the HER and OER catalytic performance of the sample in an alkaline-saturated electrolyte. The electrocatalytic performance and active surface area of the material were tested and evaluated by linear sweep voltammetry and cyclic sweep voltammetry, respectively. Among them, the scan rate of linear sweep voltammetry was 1 mV/S, HER was in the range of 0~−0.9 V vs. RHE, OER was in the range of 0.9~1.8 V vs. RHE; the scan rate of cyclic sweep voltammetry was 10, 20, 30, 40, 50, and 60 mV/s, HER in the range of −0.12~−0.22 V vs. RHE, OER in the range of 1.0~−1.1 vs. RHE, and cyclic sweep voltammetry. It can also be used to measure electrochemical double-layer capacitance. I-t curves were tested to evaluate the long-term stability of the catalysts. Electrochemical impedance spectroscopy (EIS) was tested at −0.05 V, and RHE was tested at a frequency of 0.1–100 KHz. The HER and OER potentials in an alkaline environment were calculated by the following equations.

E_RHE_ = E_Hg/HgO_ + 0.098 + 0.0591 × pH

The overall water-splitting test adopts a two-electrode system, and the synthesized Mo_4_P_3_@CoP/NF is used for the cathode and the anode, respectively. The LSV curves were determined in the alkaline electrolytes with a voltage range of 1.0 V to 2.0 V, and the stability test was performed at a constant voltage.

## 4. Conclusions

In summary, we successfully fabricated Mo_4_P_3_-supported CoP heterojunction nanoarray structures, taking nickel foam as a conductive substrate and using it as a bifunctional catalyst for water electrolysis to produce hydrogen. Experimental results show that the electrode only needs 72 and 238 mV to achieve 10 mA/cm^2^ for HER and OER in an alkaline solution, respectively. In a two-electrode system, only 1.54 V is needed to reach 10 mA/cm^2^ with high catalytic stability. It can be seen that the introduction of Mo_4_P_3_ can significantly improve the electrocatalytic activity of the materials. Among them, the construction of the Mo_4_P_3_@CoP heterointerface can induce electronic rearrangement in the interface region: adjust the electronic structure optimization in the interface area and make the interface site have better intermediate adsorption energy. In addition, in an alkaline medium, the decomposition of water molecules will seriously restrict the reaction process. It indicates that the introduction of Mo_4_P_3_ can make water molecules preferentially adsorb on the surface and easily decompose to release H*, thus reducing the energy barrier of water molecule decomposition. Therefore, the construction of the Mo_4_P_3_@CoP heterojunction can significantly optimize the adsorption energy of reactive active sites to intermediates and reduce the energy barrier of water molecule decomposition. The unique self-supporting nanoarray structure avoids the use of organic binders and ensures charge transfer between the catalytic material and the conductive collector. The above structure also facilitates adequate contact between the electrolytes and the catalytic sites, improving the catalytic efficiency. This study has important reference significance for the performance improvement of transition metal electrocatalysts.

## Data Availability

The data presented in this study are available on request from the corresponding author.

## Supplementary Materials

The following supporting information can be downloaded at: https://www.mdpi.com/article/10.3390/molecules28093647/s1. Figures S1–S17 and Tables S1–S5. References [53,54,55,56,57,58,59,60,61,62,63,64,65,66,67,68,69,70,71,72,73,74,75,76,77,78,79,80,81,82,83,84] are cited in the supplementary materials.

## Sample Availability

Samples of the compounds are available from the authors.
