# Correction: Huang et al. Constructing Molybdenum Phosphide@Cobalt Phosphide Heterostructure Nanoarrays on Nickel Foam as a Bifunctional Electrocatalyst for Enhanced Overall Water Splitting. *Molecules* 2023, *28*, 3647

**DOI:** 10.3390/molecules30244804

**Published:** 2025-12-17

**Authors:** Yingchun Huang, Hongming Chen, Busheng Zhang

**Affiliations:** Shunde Innovation School, University of Science and Technology Beijing, Foshan 528399, China

## Figure Legend

In the original publication [1], there was a mistake in the legend for Figure 6e. Figure 6e is not a capacitance measurement but rather a quantification of H_2_/O_2_. The correct legend appears below. 

Figure 6e. Faradaic efficiency of electrolytic water remeasured by drainage method.

## Addition Content in Supplementary Materials 

In response to the academic editor’s suggestions, the authors have added the specific experimental steps (including instruments, methods, pictures, etc.) for conducting quantitative analysis of the gases produced during the catalytic process. The following content has been added to the Supplementary Materials.

The two electrolytic chambers of the H-type electrolytic cell are connected to the gas pipes, and the hydrogen and oxygen generated by electrolyzing water at the current density of 100 mA cm^−2^ are imported into the measuring cylinder filled with deionized water at the liquid level of the tank. The water in the measuring cylinder is removed by the pressure of the gas so as to measure the actual gas volume generated during the catalytic process. The Faradaic efficiency of the catalyst in the electrolytic cell was calculated by comparing the measured volume of gas produced with the amount of transferred charge recorded by the electrochemical workstation. We have re-verified whether the experimental result is accurate, and the result is still similar to Figure 6e. At present, many teams are using the drainage method to calculate Faradaic efficiency. Using a gas chromatograph to measure Faradaic efficiency is very meaningful, and we will pay attention to using this method in our future work. 

The authors state that the scientific conclusions are unaffected. This correction was approved by the Academic Editor. The original publication has also been updated.
