# Supplementary material for: Constructing Molybdenum Phosphide@Cobalt Phosphide Heterostructure Nanoarrays on Nickel Foam as a Bifunctional Electrocatalyst for Enhanced Overall Water Splitting
Source: Molecules. 2023 Apr 22;28(9):3647. doi: 10.3390/molecules28093647 (PMC10180104; doi:10.3390/molecules28093647)
Supplement: Supplementary file 1 [file molecules-28-03647-s001.zip › molecules-2343332-supplementary.pdf]

Supporting Information

Constructing Molybdenum Phosphide@Cobalt Phosphide Heterostructure Nanoarrays on Nickel Foam as a Bifunctional Electrocatalyst for Enhanced Overall Water Splitting

Yingchun Huang *, Hongming Chen and Busheng Zhang

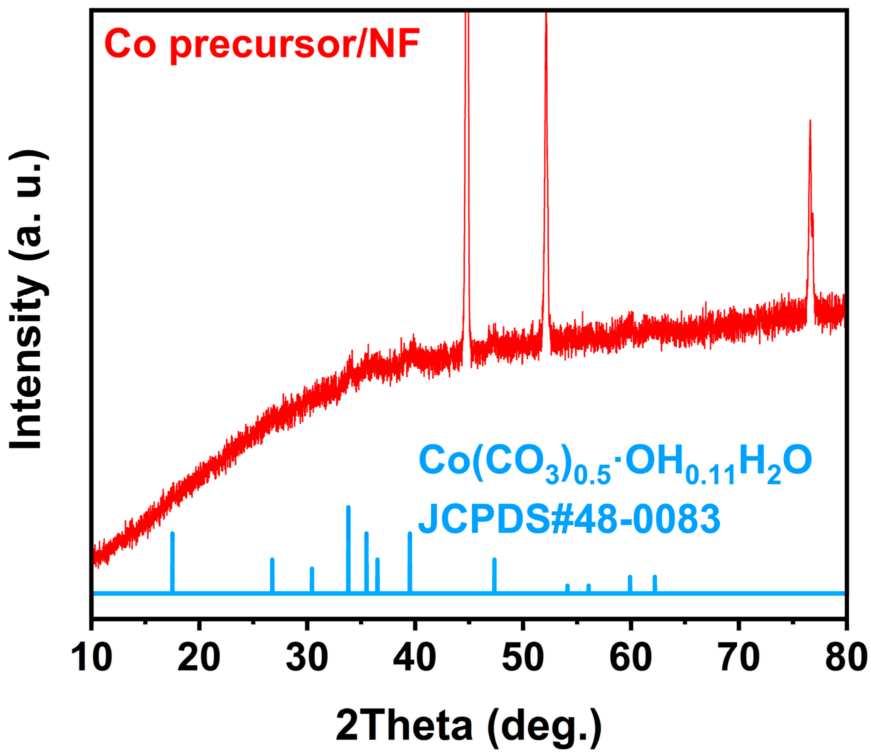

Fig. S1. XRD pattern of Co precursor/NF.

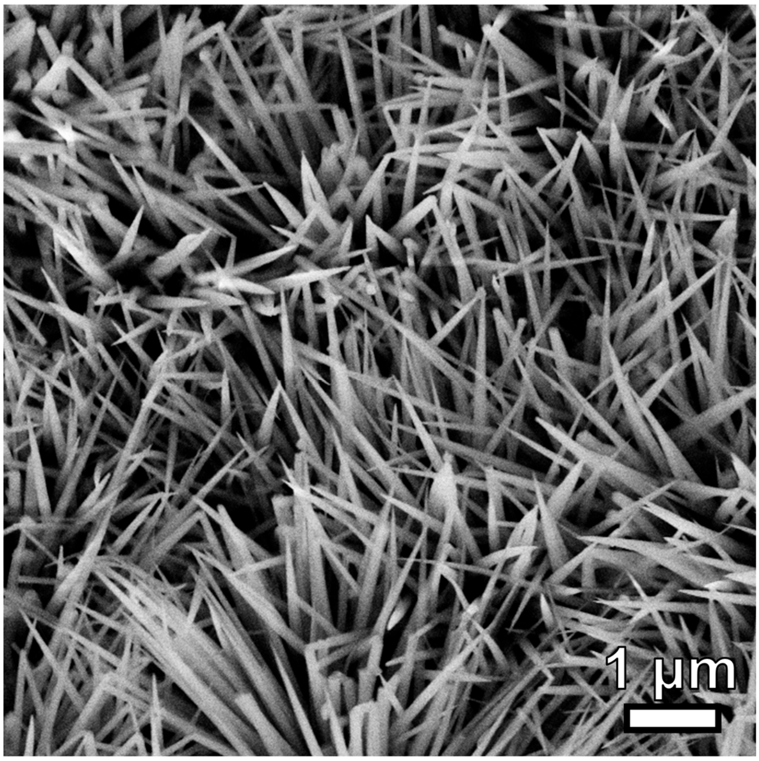

Fig. S2. Scanning electron microscopy (SEM) image of Co precursor/NF.

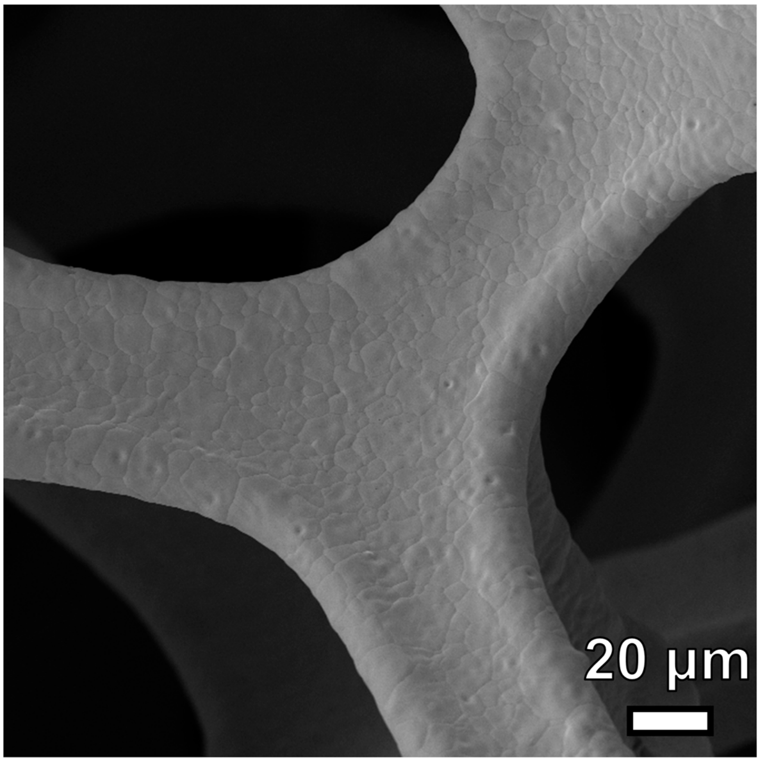

Fig. S3. Scanning electron microscopy (SEM) image of Ni poam(NF).

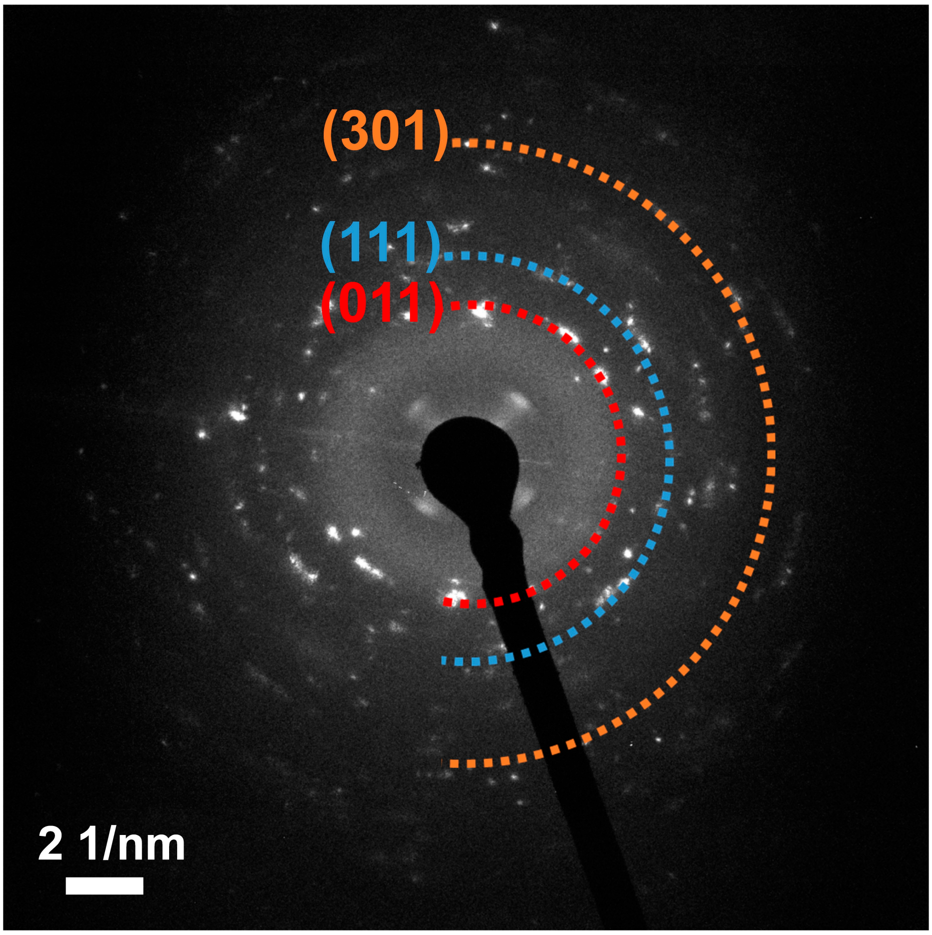

Fig. S4. Selected area electron diffraction (SAED) pattern of Mo_4P_3@CoP/NF.

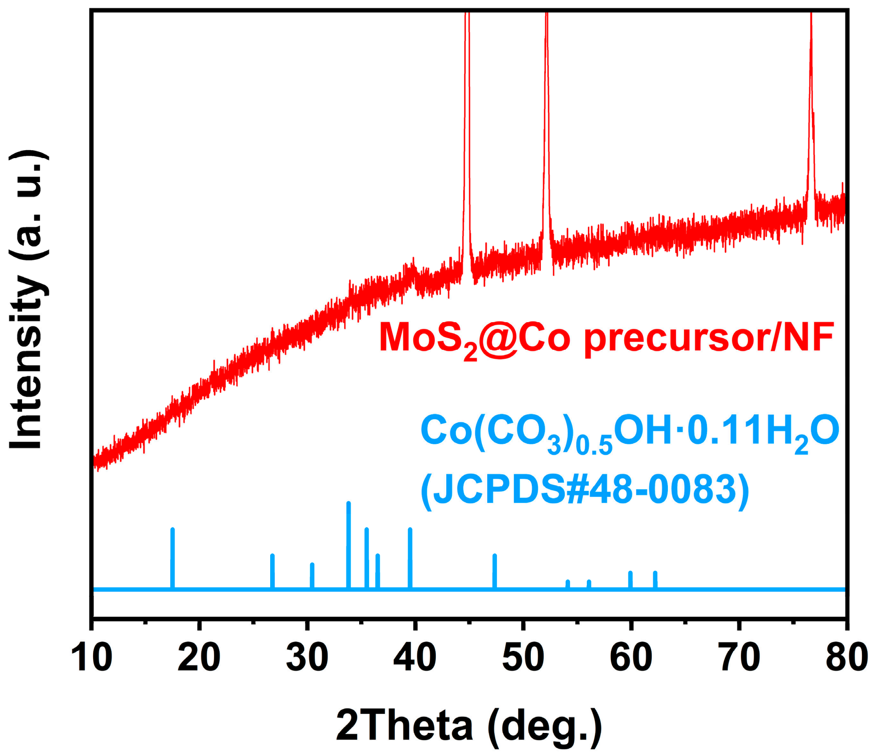

Fig. S5. XRD pattern of MoS_2@Co precursor/NF.

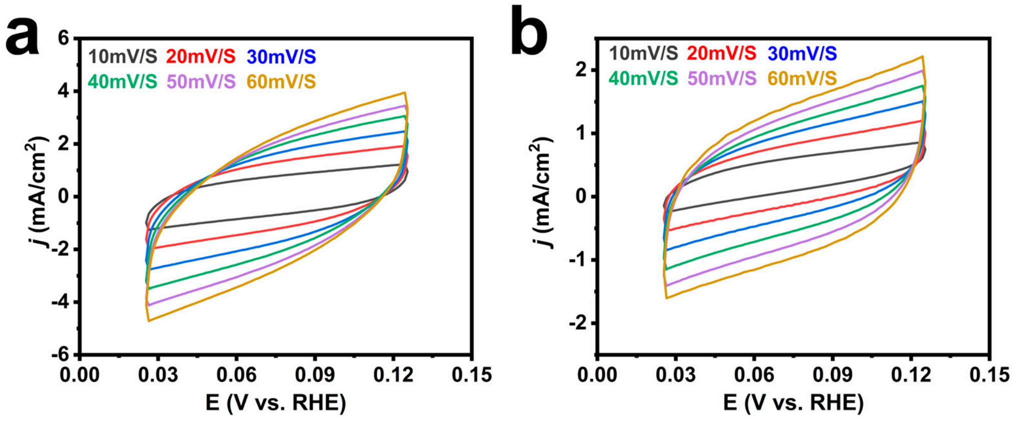

Fig. S6. Cyclic voltammograms of (a) Mo_4P_3@CoP/NF and (b) CoP/NF obtained in -0.12~ -0.22 V vs. RHE region at scan rates of 10, 20, 30, 40, 50, and 60 mV/s, respectively.

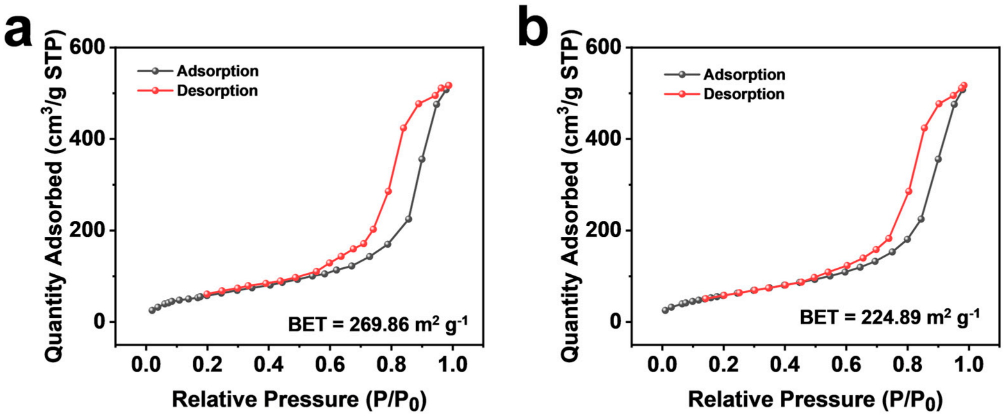

Fig. S7. BET specific surface area and average pore sizes of Mo_4P_3@CoP/NF and CoP/NF.

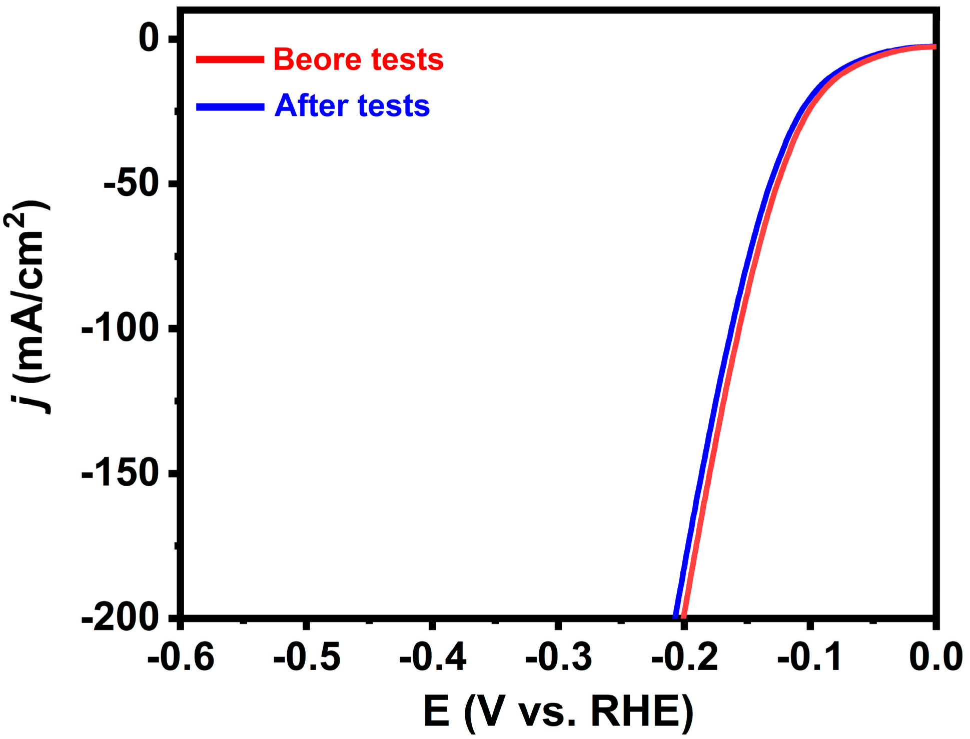

Fig. S8. LSV curve of Mo_4P_3@CoP/NF electrode after chronoamperometry test in 1 M KOH.

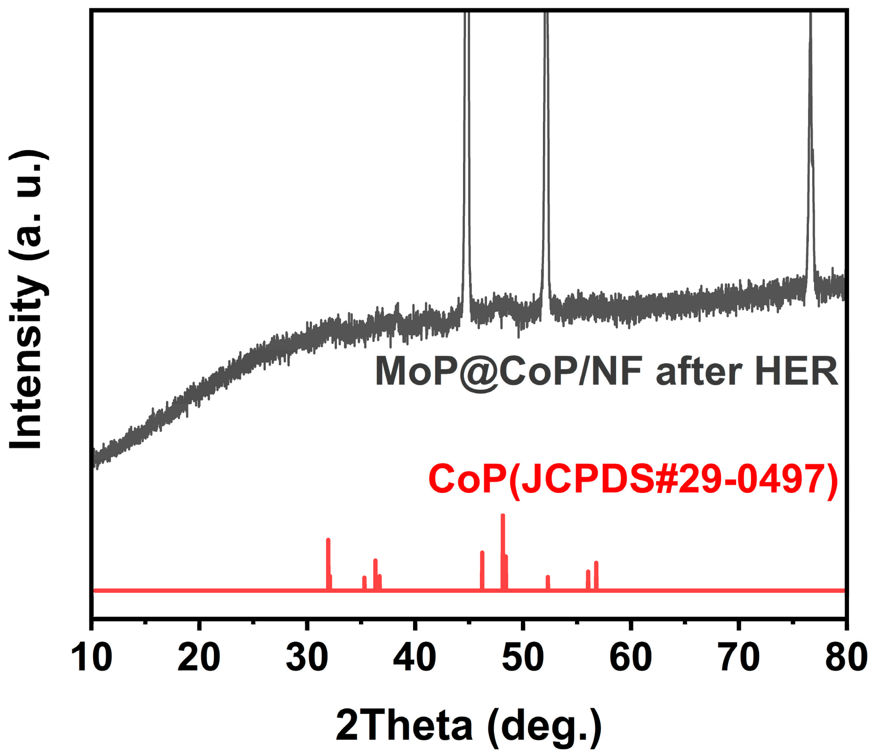

Fig. S9. XRD pattern of Mo_4P_3@CoP/NF after HER(JCPDS#29-0497).

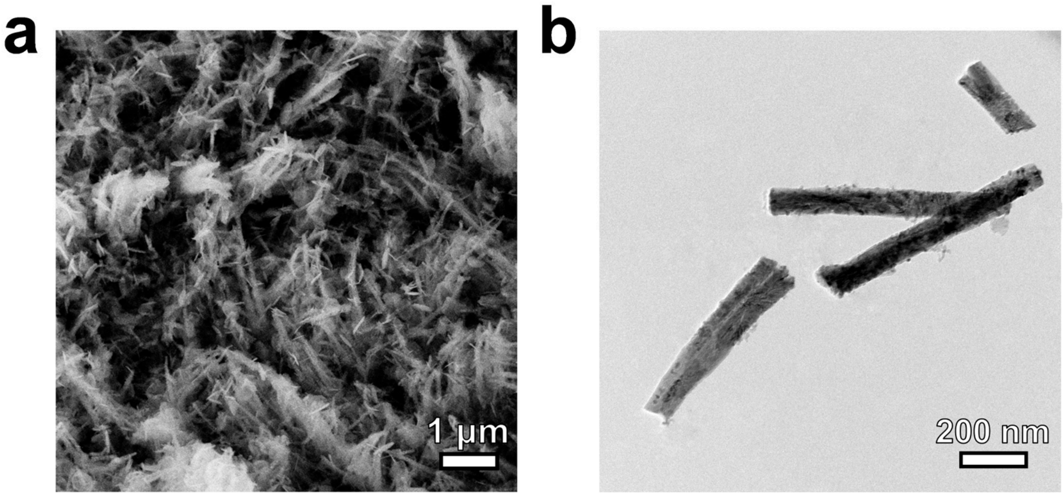

Fig. S10. (a) Scanning electron microscopy (SEM) image and (b) transmission electron microscopy (TEM) image of Mo_4P_3@CoP/NF after HER.

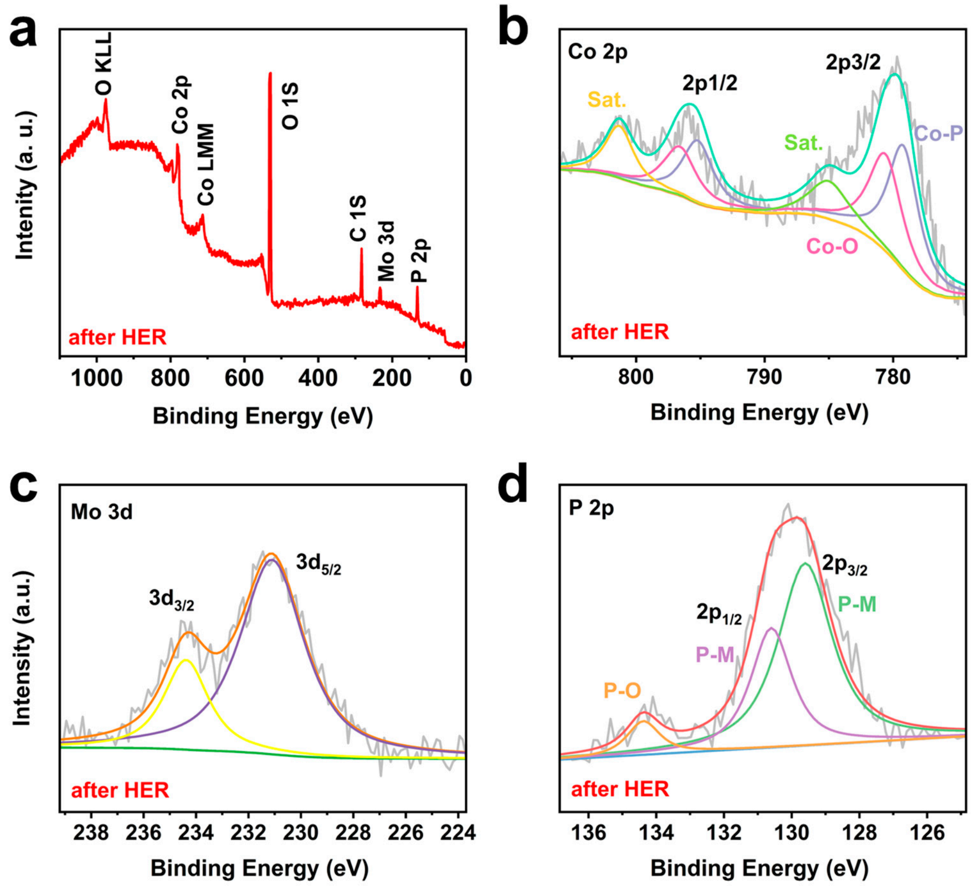

Fig. S11. (a) Full XPS spectra of Mo_4P_3@CoP/NF after HER. High-resolution (b) Co 2p, (c) Mo 3d and (d) P 2p XPS spectra of Mo_4P_3@CoP/NF after HER.

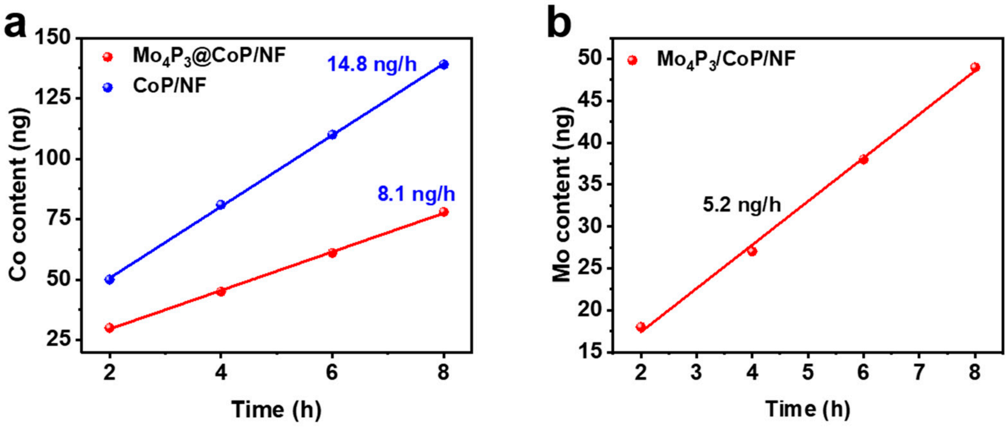

Fig. S12. (a) Co and (b) Mo contents in electrolytes after the chronopotentiometric test at 10 mA cm^{-2}.

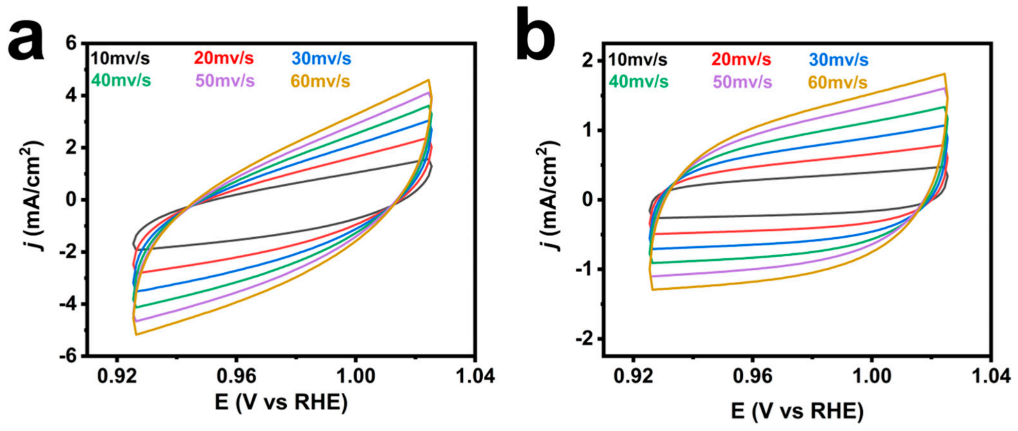

Fig. S13. Cyclic voltammograms of (a) Mo_4P_3@CoP/NF and (b) CoP/NF obtained in 1.0~ -1.1 V vs. RHE region at scan rates of 10, 20, 30, 40, 50, and 60 mV/s, respectively.

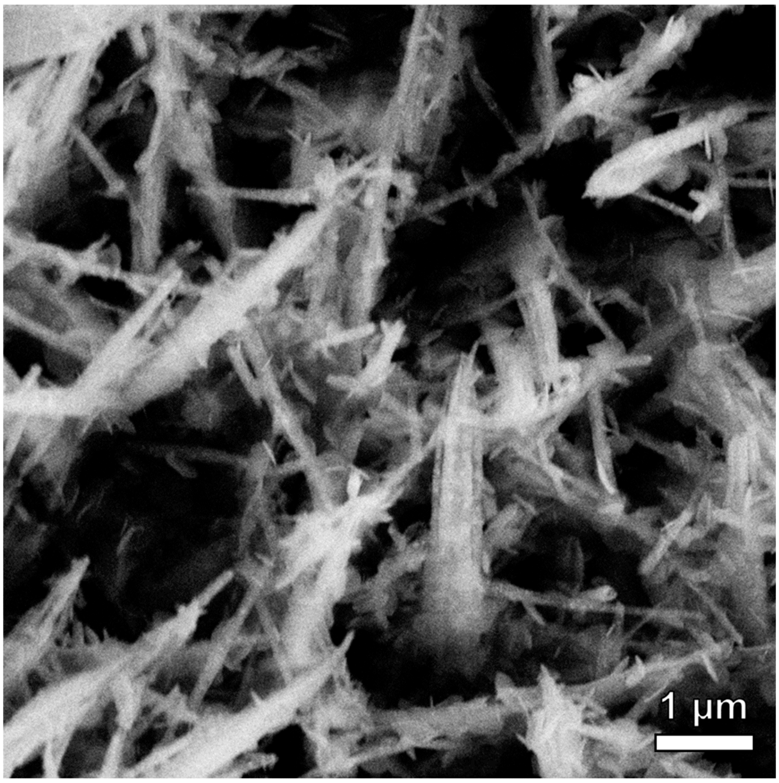

Fig. S14. SEM pattern of Mo_4P_3@CoP/NF after OER.

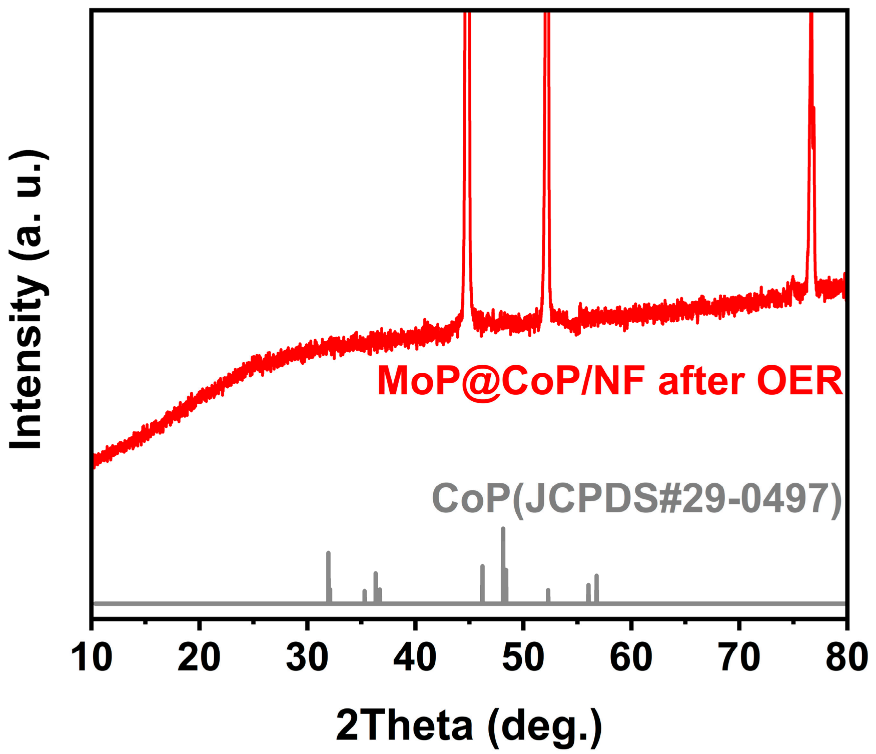

Fig. S15. (a) XRD pattern of Mo_4P_3@CoP/NF after OER(JCPDS#29-0497).

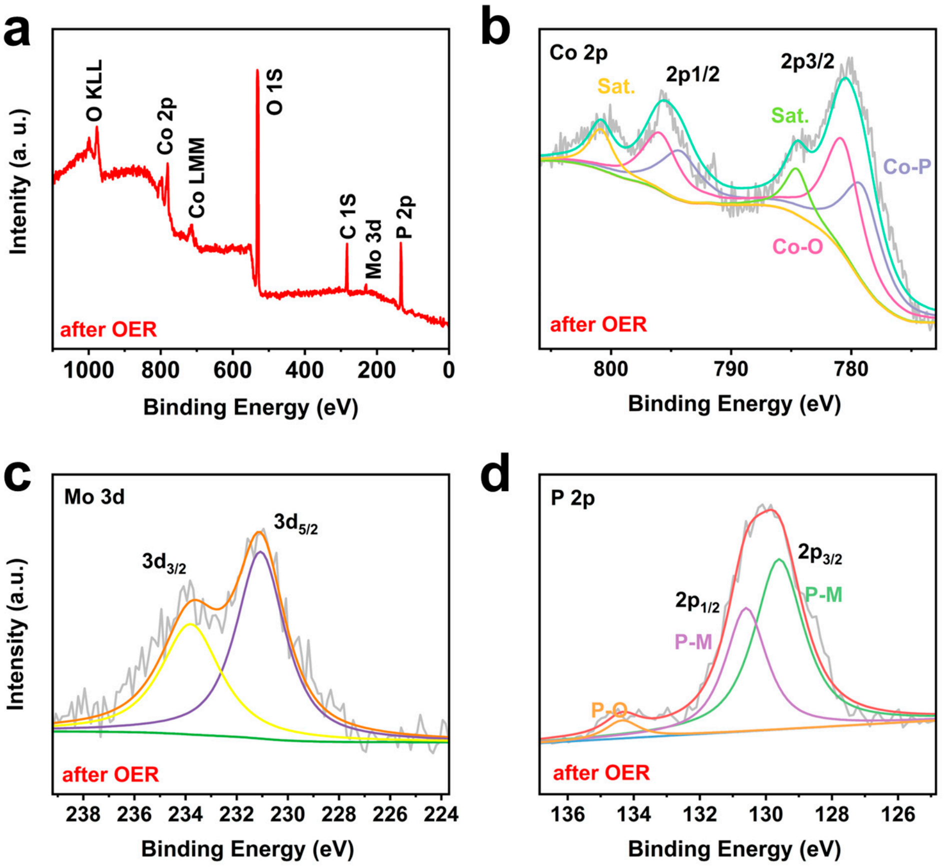

Fig. S16. (a) full XPS spectra of Mo_4P_3@CoP/NF after OER. High-resolution (b) Co 2p, (c) Mo 3d and (d) P 2p XPS spectra of Mo_4P_3@CoP/NF after OER.

The specific experimental steps (including instruments, methods, pictures, etc.) for conducting quantitative analysis of the gases produced during the catalytic process are as follows

The two electrolytic chambers of the H-type electrolytic cell are connected to the gas pipes, and the hydrogen and oxygen generated by electrolyzing water at the current density of 100 mA cm^{-2} are imported into the measuring cylinder filled with deionized water at the liquid level of the tank. The water in the measuring cylinder is removed by the pressure of the gas so as to measure the actual gas volume generated during the catalytic process. The Faradaic efficiency of the catalyst in the electrolytic cell was calculated by comparing the measured volume of gas produced with the amount of transferred charge recorded by the electrochemical workstation. We have re-verified whether the experimental result is accurate, and the result is still similar to Figure 6e. At present, many teams are using the drainage method to calculate Faradaic efficiency. Using a gas chromatograph to measure Faradaic efficiency is very meaningful, and we will pay attention to using this method in our future work.
Below are photos of the data and experiments we retested.

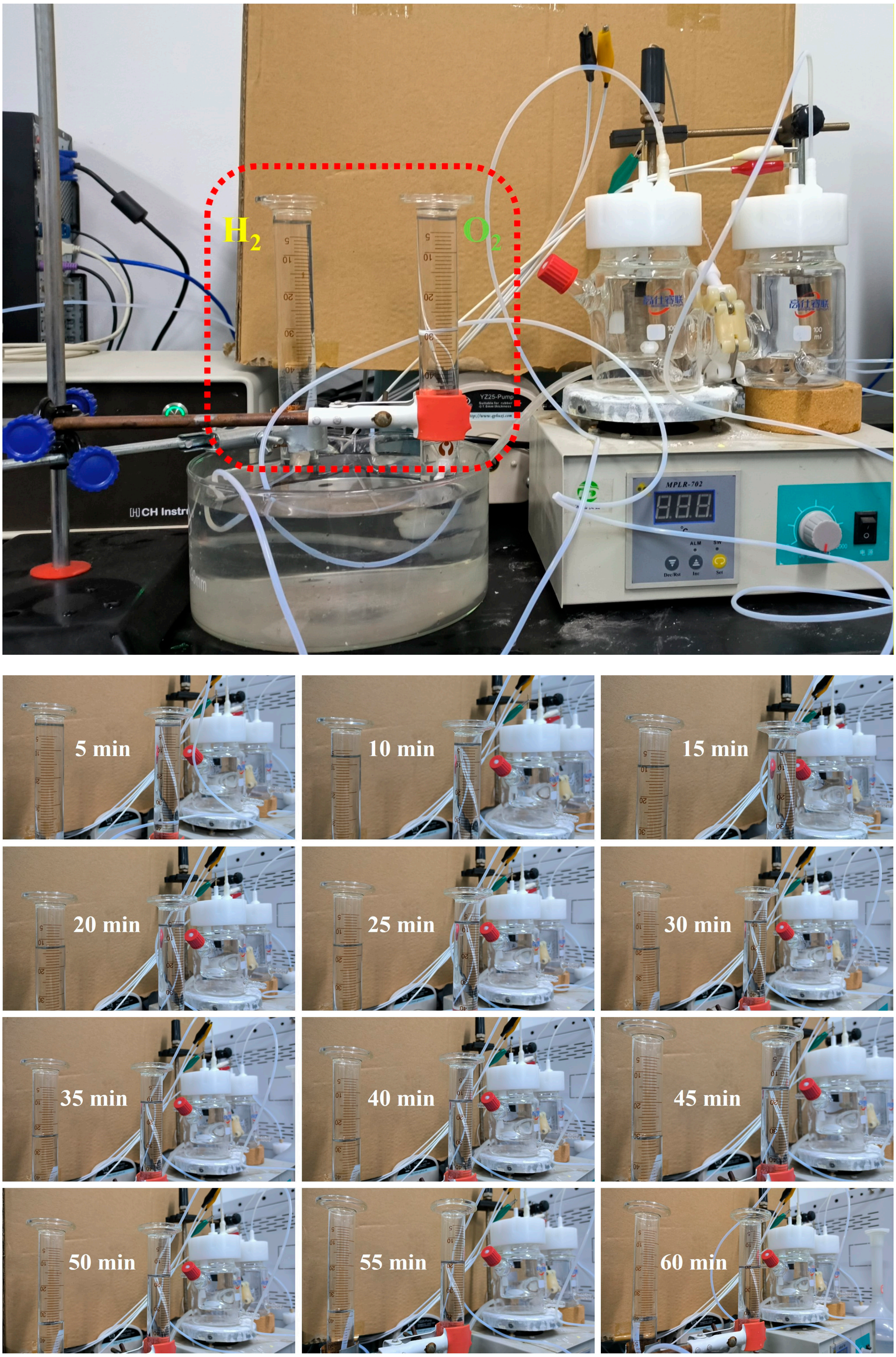

Figure S17 Device diagram for measuring Faradaic efficiency by drainage method.

Table S1. Co contents in electrolytes after the chronopotentiometric test at 10 mA cm^{-2}.

Catalysts	2 h (ng)	4 h (ng)	8 h (ng)	10 h (ng)
**Mo_4P_3@CoP/NF**	**30**	**45**	**61**	**78**
CoP/NF	50	81	110	139

Table S2. Mo contents in electrolytes after the chronopotentiometric test at 10 mA cm^{-2}.

Catalysts	2 h (ng)	4 h (ng)	8 h (ng)	10 h (ng)
**Mo_4P_3@CoP/NF**	**18**	**27**	**38**	**49**

Table S3. The OER performance of Ce-$Co(OH)_2$/CoP/NF electrode with other reported self-supported electrocatalysts in 1 M KOH solution.

**Catalyst**	**Electrolyte**	**η (mV) @ 10 mA/cm^2**	**Tafel slope (mV/dec)**	**Reference**
Mn-NSG	1 M KOH	296	38	[1]
CoC2O4/Ag2C2O4	1 M KOH	260	47	[2]
$SrNb_{0.1}Co_{0.7}Fe_{0.2}O_{3-\delta}$	1 M KOH	278	56	[3]
P-CoPc@CNT	1 M KOH	300	41.7	[4]
FeCoSx-PBA	1 M KOH	266	33	[5]
N,F−FeO(OH)−CO_3−NF	1 M KOH	248	34.51	[6]
FeCoNiMo	1 M KOH	250	48.02	[7]
Mn-ZIF-67-NFs	1 M KOH	302	125	[8]

NiCo-LDH-OH	1 M KOH	317	172.85	[9]
Mo_4P_3**@CoP/NF**	**1 M KOH**	**238**	**60**	**Our work**

Table S4. The HER performance of Ce-Co(OH)$_2$/CoP/NF electrode with other reported self-supported electrocatalysts in 1 M KOH solution.

**Catalyst**	**Electrolyte**	**η (mV) @ 10 mA/cm^2**	**Tafel slope (mV/dec)**	**Reference**
MoS_2 monolayer	1 M KOH	90	94	[10]
APNEs	1 M KOH	101	94.3	[11]
CoFeNiP/NF	1 M KOH	104	108	[12]
Mo($FeNi_3Mo_{0.40}$)	1 M KOH	112	109	[13]
CNN-500	1 M KOH	127	120	[14]
$FeMo_2S_4$	1 M KOH	78	118	[15]
Ni-Ce-Pr-Ho/NF	1 M KOH	78	121.6	[16]
Ni-VN-V_2O_3/NC	1 M KOH	76	142	[17]
Co-WS_2/P-$WO_{2.9}$	1 M KOH	146	86	[18]
Mo_4P_3@**CoP/NF**	**1 M KOH**	**72**	**89**	**Our work**

Table S5. The overall water splitting performance of Ce-Co(OH)$_2$/CoP/NF electrode with other reported bifunctional electrocatalysts in 1 M KOH solution.

Catalyst	Electrolyte	E (V) @ 10 mA/cm^2	Reference
$NiRuO_{2-x}$	1 M KOH	1.6	[19]
Fe_3N_4N@Co@CoFe	1 M KOH	1.59	[20]
(Ni3S2-MoS2)@TiO2	1 M KOH	1.56	[21]
SnO2@MoS2/NF	1 M KOH	1.57	[22]
Mo_4P_3-Mo2C/NPC	1 M KOH	1.55	[23]
La-$CoMo_4P_3$	1 M KOH	1.56	[24]
NiFe-Pi/P	1 M KOH	1.57	[25]
Co-N-C/CP	1 M KOH	1.56	[26]
IrNiTa (INT) MG	1 M KOH	1.58	[27]
Co/CoMoN/NF	1 M KOH	1.56	[28]
Fe_3O_4/RuO_2-C	1 M KOH	1.595	[29]
SSM/CoOP	1 M KOH	1.57	[30]
NiFeMo/NF	1 M KOH	1.62	[31]
$Mo_2TiC_2T_x$	1 M KOH	1.57	[32]
Mo_4P_3**@CoP/NF**	**1 MKOH**	**1.54**	**Our work**

Reference

[1] X. Bai, L. Wang, B. Nan, T. Tang, X. Niu, J. Guan, Atomic manganese coordinated to nitrogen and sulfur for oxygen evolution, Nano Research, 15 (2022) 6019-6025.

[2] S. Ghosh, A. Mondal, G. Tudu, S. Ghosh, H.V.S.R.M. Koppisetti, H.R. Inta, D. Saha, V. Mahalingam, Efficient Electrochemical Reconstruction of a Cobalt- and Silver-Based Precatalytic Oxalate Framework for Boosting the Alkaline Water Oxidation Performance, ACS Sustainable Chemistry & Engineering, 10 (2022) 7265-7276.

[3] R. Hu, M. Zhao, H. Miao, F. Liu, J. Zou, C. Zhang, Q. Wang, Z. Tian, Q. Zhang, J. Yuan, Rapidly reconstructing the active surface of cobalt-based perovskites for alkaline seawater splitting, Nanoscale, 14 (2022) 10118-10124.

[4] Y. Liu, S. Zhang, C. Jiao, H. Chen, G. Wang, W. Wu, Z. Zhuo, J. Mao, Axial Phosphate Coordination in Co Single Atoms Boosts Electrochemical Oxygen Evolution, Adv Sci (Weinh), 10 (2023) 2206107.

[5] M. Lu, L. An, J. Yin, J. Jin, R. Yang, B. Huang, Y. Hu, Y.-Q. Zhao, P. Xi, Electronic engineering of amorphous Fe–Co–S sites in hetero-nanoframes for oxygen evolution and flexible Al–air batteries, Journal of Materials Chemistry A, 10 (2022) 19757-19768.

[6] J.Q. Lv, X. Chen, Y. Chang, Y.G. Li, H.Y. Zang, N, F Codoped FeOOH Nanosheets with Intercalated Carbonate Anions Rich in Oxygen Defects for Enhanced Alkaline Electrocatalytic Water Splitting, ACS Appl Mater Interfaces, 14 (2022) 52877-52885.

[7] Y. Mei, Y. Feng, C. Zhang, Y. Zhang, Q. Qi, J. Hu, High-Entropy Alloy with Mo-Coordination as Efficient Electrocatalyst for Oxygen Evolution Reaction, ACS Catalysis, 12 (2022) 10808-10817.

[8] S.S. Selvasundarasekar, T.K. Bijoy, S. Kumaravel, A. Karmakar, R. Madhu, K. Bera, S. Nagappan, H.N. Dhandapani, G.A.M. Mersal, M.M. Ibrahim, D. Sarkar, S.M. Yusuf, S.C. Lee, S. Kundu, Effective Formation of a Mn-ZIF-67 Nanofibrous Network via Electrospinning: An Active Electrocatalyst for OER in Alkaline Medium, ACS Appl Mater Interfaces, 14 (2022) 46581-46594.

[9] H. Yang, Z. Zhou, H. Yu, H. Wen, R. Yang, S. Peng, M. Sun, L. Yu, Alkali treatment of layered double hydroxide nanosheets as highly efficient bifunctional electrocatalysts for overall water splitting, J Colloid Interface Sci, 636 (2023) 11-20.

[10] J. Qu, Y. Li, F. Li, T. Li, X. Wang, Y. Yin, L. Ma, O.G. Schmidt, F. Zhu, Direct Thermal Enhancement of Hydrogen Evolution Reaction of On-Chip Monolayer MoS_2, ACS Nano, 16 (2022) 2921-2927.
[11] F. Xiao, L. Li, W. Cui, Y. Zhang, C. Zhan, W. Xiao, Aligned porous nickel electrodes fabricated via ice templating with submicron particles for hydrogen evolution in alkaline water electrolysis, Journal of Power Sources, 556 (2023) 232441.
[12] M. Wang, Y. Chen, T. Li, Controllable preparation of nickel phosphide using iron and cobalt as electrocatalyst for hydrogen evolution reaction in alkaline media, Materials Today Chemistry, 24 (2022) 100914.
[13] M. Rafei, X. Wu, A. Piñeiro Garcia, V. Miranda la Hera, T. Wågberg, E. Gracia-Espino, Non-Stoichiometric NiFeMo Solid Solutions; Tuning the Hydrogen Adsorption Energy via Molybdenum Incorporation, Advanced Materials Interfaces, 9 (2022) 2201214.
[14] Y. Yan, Q. Ma, F. Cui, J. Zhang, T. Cui, Carbon onions coated Ni/NiO nanoparticles as catalysts for alkaline hydrogen evolution reaction, Electrochimica Acta, 430 (2022) 141090.
[15] Z.Y. Tian, X.Q. Han, J. Du, Z.B. Li, Y.Y. Ma, Z.G. Han, Bio-Inspired $FeMo_2S_4$ Microspheres as Bifunctional Electrocatalysts for Boosting Hydrogen Oxidation/Evolution Reactions in Alkaline Solution, ACS Appl Mater Interfaces, 15 (2023) 11853-11865.
[16] W. Liu, W. Tan, H. He, Y. Peng, Y. Chen, Y. Yang, One–step electrodeposition of Ni–Ce–Pr–Ho/NF as an efficient electrocatalyst for hydrogen evolution reaction in alkaline medium, Energy, 250 (2022) 123831.
[17] H.-H. Zou, W.-Q. Li, C.-H. Song, L.-M. Cao, X.-F. Zhang, X.-Y. Zhu, Z.-Y. Du, J. Zhang, S.-L. Zhong, C.-T. He, Disclosing the active integration structure and robustness of a pseudo-tri-component electrocatalyst toward alkaline hydrogen evolution, Journal of Energy Chemistry, 72 (2022) 210-216.
[18] Y. Wang, S. Yun, J. Shi, Y. Zhang, J. Dang, C. Dang, Z. Liu, Y. Deng, T. Yang, Defect engineering tuning electron structure of biphasic tungsten-based chalcogenide heterostructure improves its catalytic activity for hydrogen evolution and triiodide

reduction, J Colloid Interface Sci, 625 (2022) 800-816.

[19] X. Chen, J. Song, Y. Xing, Y. Qin, J. Lin, X. Qu, B. Sun, S. Du, D. Shi, C. Chen, D. Sun, Nickel-decorated RuO_2 nanocrystals with rich oxygen vacancies for high-efficiency overall water splitting, J Colloid Interface Sci, 630 (2023) 940-950.

[20] Z. Cui, X. Liang, P. Wang, P. Zhou, Q. Zhang, Z. Wang, Z. Zheng, Y. Liu, Y. Dai, B. Huang, In situ integration of Fe_3N@Co_4N@CoFe alloy nanoparticles as efficient and stable electrocatalyst for overall water splitting, Electrochimica Acta, 395 (2021) 139218.

[21] D. Guo, Z. Wan, G. Fang, M. Zhu, B. Xi, A Tandem Interfaced (Ni_3S_2-MoS_2@TiO_2 Composite Fabricated by Atomic Layer Deposition as Efficient HER Electrocatalyst, Small, 18 (2022) 2201896.

[22] W. He, L. Wang, H. Zhang, S. Gao, W. Yu, D. Yin, X. Dong, SnO_2@MoS_2 heterostructures grown on nickel foam as highly efficient bifunctional electrocatalyst for overall water splitting in alkaline media, Journal of Alloys and Compounds, 938 (2023) 168678.

[23] E. Jiang, J. Li, X. Li, A. Ali, G. Wang, S. Ma, P. Kang Shen, J. Zhu, Mo_4P_3-Mo_2C quantum dot heterostructures uniformly hosted on a heteroatom-doped 3D porous carbon sheet network as an efficient bifunctional electrocatalyst for overall water splitting, Chemical Engineering Journal, 431 (2022) 133719.

[24] L. Li, K. Chao, X. Liu, S. Zhou, Construction of La decorated $CoMo_4P_3$ composite and its highly efficient electrocatalytic activity for overall water splitting in alkaline media, Journal of Alloys and Compounds, 941 (2023) 168952.

[25] W. Li, M. Chen, Y. Lu, P. Qi, G. Liu, Y. Zhao, H. Wu, Y. Tang, One-pot electrodeposition synthesis of NiFe-phosphate/phosphide hybrid nanosheet arrays for efficient water splitting, Applied Surface Science, 598 (2022) 153717.

[26] X. Li, F. Duan, X. Lu, Y. Gang, W. Zheng, Y. Lin, L. Chen, Y. Dan, X. Cheng, Surface engineering of flower-like Co-N-C on carbon paper for improved overall water splitting, Journal of Alloys and Compounds, 935 (2023) 168128.

[27] D. Liu, Z. Song, S. Cheng, Y. Wang, A. Saad, S. Deng, J. Shen, X. Huang, X. Cai, P. Tsiakaras, Mesoporous IrNiTa metal glass ribbon as a superior self-standing

bifunctional catalyst for water electrolysis, Chemical Engineering Journal, 431 (2022) 134210.

[28] H. Ma, Z. Chen, Z. Wang, C.V. Singh, Q. Jiang, Interface Engineering of Co/CoMoN/NF Heterostructures for High-Performance Electrochemical Overall Water Splitting, Adv Sci (Weinh), 9 (2022) 2105313.

[29] A. Shekhawat, R. Samanta, S. Barman, MOF-Derived Porous Fe_3O_4/RuO_2-C Composite for Efficient Alkaline Overall Water Splitting, ACS Applied Energy Materials, 5 (2022) 6059-6069.

[30] Q. Tan, R. Xiao, X. Yao, T. Xiong, J. Li, Y.-w. Hu, Y. Huang, M.S. Balogun, Non-oxygen anion-regulated in situ cobalt based heterojunctions for active alkaline hydrogen evolution catalysis, Chemical Engineering Journal, 433 (2022) 133514.

[31] Z. Wang, H. Chen, J. Bao, Y. Song, X. She, G. Lv, J. Deng, H. Li, H. Xu, Amorphized core–shell NiFeMo electrode for efficient bifunctional water splitting, Applied Surface Science, 607 (2023) 154803.

[32] S.A. Zahra, M. Waqas Hakim, M. Adil Mansoor, S. Rizwan, Two-dimensional double transition metal carbides as superior bifunctional electrocatalysts for overall water splitting, Electrochimica Acta, 434 (2022) 141257.
